# Efficacy of a Novel Intraoperative Engineered Sharps Injury Prevention Device: Pilot Usability and Efficacy Trial

**DOI:** 10.2196/19729

**Published:** 2020-11-27

**Authors:** Hillary Jenny, Maria Reategui Via y Rada, Pooja Yesantharao, Helen Xun, Richard Redett, Justin Michael Sacks, Robin Yang

**Affiliations:** 1 Department of Plastic & Reconstructive Surgery Johns Hopkins Hospital Baltimore, MD United States; 2 Geisel School of Medicine at Dartmouth Hanover, NH United States

**Keywords:** needlestick injuries, sharps injuries, needles, safety, perioperative safety, intraoperative safety, quality improvement, case-control studies, tertiary care centers, plastic surgery, surgeons

## Abstract

**Background:**

The American College of Surgeons reports 88,320 intraoperative needlestick injuries (NSIs) per year, resulting in US $376 to US $2456 in costs per NSI. Engineered sharps injury prevention (ESIP) devices protect against NSIs. To our knowledge, no study has been published to date to demonstrate clinical effectiveness of an intraoperative ESIP device. Operative Armour is a wearable arm cuff that can be donned during surgical closure to allow surgeons to keep a suture pack and sharps protection container on their forearm.

**Objective:**

We characterize Operative Armour’s ESIP device effectiveness in a tertiary hospital, hypothesizing that this device will decrease NSI risk by decreasing behaviors associated with NSIs: needle passing and handling.

**Methods:**

A prospective case-control study was conducted with institutional review board quality improvement designation in which authors observed skin closures of plastic surgery procedures. To ensure accuracy, one surgeon was observed at a time. Control surgeries were purely observational; intervention cases involved surgeon use of the device during skin closure. Outcomes of interest included needle passing, needle handling, lost needles, and loaded waiting needles.

**Results:**

Surgeons were observed in 50 control and 50 intervention cases. Operative Armour eliminated needle passing during skin closure. One NSI occurred in one control case; no NSIs were observed in intervention cases (*P*=.36). The mean number of loaded and unprotected waiting needles was also significantly decreased in the intervention group from 2.3 to 0.2 (*P*<.001). Furthermore, a multivariable linear regression established that Operative Armour significantly decreased the number of needle adjustments by hand per stitch observed (*F*_4, 21.68_=3.72; *P*=.01). In fact, needle adjustments by hand decreased overall (1 adjustment per 10 stitches vs 1 adjustment per 5 stitches, *P*=.004), and adjustments occurred half as frequently with use of Operative Armour in free flap reconstruction (1 adjustment per 10 stitches vs 1 adjustment per 5 stitches, *P*=.03) and a quarter as frequently in other breast reconstruction cases such as mastopexy (1 adjustment per 20 stitches vs 1 adjustment per 5 stitches, *P*=.002).

**Conclusions:**

Operative Armour effectively functions as an ESIP device by decreasing intraoperative needle passing and handling. Although sample size prohibits demonstrating a decrease in NSIs during observed cases, by decreasing behaviors that drive NSI risk, we anticipate an associated decrease in NSIs with use of the device.

## Introduction

Needlestick injuries (NSIs) have been estimated to occur at a rate of 1.55% per surgeon per operation [1]. Other studies estimate that NSIs occur in 1.7% to 15% of all procedures [2], with the American College of Surgeons reporting 88,320 NSIs a year [3]. Surgeons or their first assistant are at the highest risk of injury, accounting for 59% of NSIs, followed by scrub personnel (19%), anesthesiologists (6%), and circulating nurses (6%) [2]. The most common cause of sharps injuries in surgeons are suture needles, of which over half occur during suturing of fascia or muscles [4]. Up to 16% of injuries have been found to occur while passing sharp instruments hand to hand [2]. These needle handoffs occur frequently in the operating room as needles are loaded by the scrub tech, passed off to the surgeon, and handed back to the tech once the stitch is thrown.

NSIs pose a significant health risk to employees. After NSI from an infected source, the risk of acquiring hepatitis B virus is between 2% to 40% [5], hepatitis C virus 3% to 10%, and HIV 0.2% to 0.5% [6]. In addition to the health risk these NSIs present, they pose a significant financial burden: each NSI costs anywhere from US $376 to US $2456 for an estimated yearly national cost from US $33 million to US $2 billion [7]. Under these circumstances, there is a pressing need to reduce needlestick injuries, particularly in the surgical setting [8].

**Figure 1:**
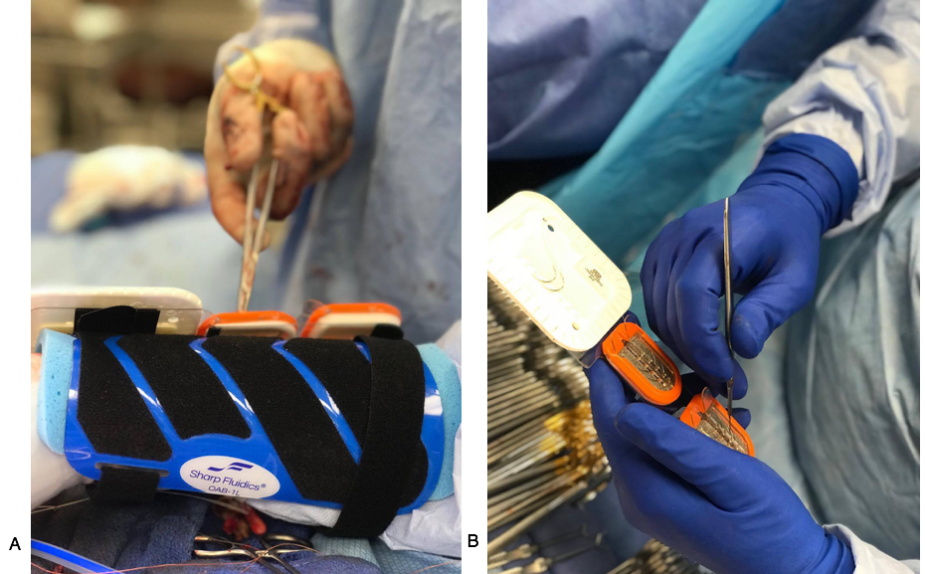
Operative Armour is a wearable arm cuff allowing the surgeon to reload and protect needles, reducing the number of handoffs between the surgeon and scrub technician. (A) The surgeon wears the arm cuff (blue), upon which the Operative Armour sharps container storage (orange) and any suture pack (white) can be placed using adhesives and Velcro. Here, the surgeon is sliding the needle into the storage container. (B) Following exchange of the needle pack, the scrub technician performs the needle count of the needles protected in the sharps storage container.

Operative Armour is a wearable arm cuff that allows surgeons to keep a suture pack and a sharps protection container on their forearm. The arm cuff is worn by the surgeon on their nondominant forearm, strapped on by adjustable Velcro. The surgeon positions a suture pack and the Operative Armour sharps protection container on the forearm cuff through adhesives and Velcro (Figure 1A). The surgeon is then able to use their needle driver to directly pick up needles from the cuff. The surgeon then stores the needle by sliding the needle into the sharps protection container, which features shelves that trap the unprotected needle. When the surgeons reload, the suture pack and sharps protection container are exchanged for a new set, allowing the scrub technician to perform the needle count with the returned Operative Armour sharps protection container (Figure 1B).

By allowing the surgeon to have the suture pack and needle storage container on their own forearm, passing unprotected needles between surgical technician and surgeon will no longer be needed. Needle handling is also decreased as the surgeon can load the needle themselves and does not need to protect the needle after suturing as it is no longer passed off to the surgical technician. We aim to characterize Operative Armour’s effectiveness as an intraoperative sharps protection device in a tertiary hospital, hypothesizing that this device will decrease NSI risk by decreasing behaviors associated with NSIs: needle passing and handling.

## Methods

### Study Design

A prospective case-control study was conducted with Johns Hopkins School of Medicine institutional review board quality improvement designation (IRB00207584, approved April 24, 2019) in which authors observed skin and soft tissue closures of plastic surgery procedures. Data were collected from August 1 to December 1, 2019. Cases were included if they were plastic surgery procedures with long closures (>10 cm total length) and multiple surgeons (2 or more). These surgeries included abdominal hernia repairs, panniculectomies, breast reconstruction with free tissue transfer, breast reconstruction with implants, and breast reductions and mastopexies for both macromastia and breast reconstructive purposes. To ensure accuracy, one surgeon was observed at a time.

### Case/Control Grouping

Control surgeries were purely observational. Study outcomes were observed and noted without intervention. Case surgeries (intervention surgeries) involved surgeon donning and using Operative Armour during skin closure. For analysis, procedures were grouped into 3 cohorts: abdominal surgery (abdominal hernia repairs, panniculectomies), breast reconstruction (free tissue transfer or implant-based), and mastopexy/breast reduction or revision (mastopexies, reduction mammaplasties, and breast reconstruction revision procedures).

### Outcomes

The primary outcome of interest was needle passing, which was defined as the needle being passed hand to hand between two people. Secondary outcomes included needle handling; needles that were dropped, temporarily misplaced, or permanently lost; loaded waiting needles; and needlestick injury occurrence. Needle handling was defined as surgeon hand contact with the needle. Dropped needles were dropped on the floor during any part of the suturing process, either realized in the moment or later during count. Temporarily misplaced needles were needles that were temporarily absent from the needle count and caused significant search and recount efforts before they were found; these were typically either dropped or misplaced in a less visible area of the sterile field. Permanently lost needles were never found. Loaded waiting needles were defined as needles that were loaded on a needle driver with the sharp edge exposed, waiting to be used for suturing. The washout period during acclimation to the device was determined on a case by case basis, with the observer and surgeon coming to an agreement that the surgeon is acclimated and suturing with a similar speed and facility with Operative Armour as without. Cases were observed by nonscrubbed authors HJ, MLR, PY, and HX from within the operating room at a safe distance to maintain surgical field sterility. All observers were trained in outcome definition and key data points for observation and documentation.

### Statistical Analysis

All statistical analyses were completed using Stata version 13 (StataCorp LLC). Schapiro-Wilk testing was used to determine normality of all continuous variables. Descriptive statistics by case type were calculated using analyses of variance and chi square analyses where appropriate. Multiple linear regression was used to analyze the impact of intervention (Operative Armor vs control) on the number of needle adjustments, after adjusting for surgeon and case-level covariates. The 2-tailed threshold for statistical significance was set at an alpha value of .05. The Bonferroni correction was used for multiple comparisons. Post hoc power analyses were completed using G*Power software (IDRE Statistical Consulting).

## Results

### Surgeon and Case Characteristics

In total, 4 attending surgeons and 13 surgical residents were observed in 50 control and 50 intervention cases (Table 1).

**Table 1:** Surgeon/case characteristics for control and Operative Armour groups.

Characteristic		Control (n=50)	Operative Armour (n=50)	*P* value
**Case type, n (%)**		**N/A^a^**	**N/A**	**.02**
	Abdominal surgery	11 (22)	8 (16)	N/A
	Breast reconstruction	26 (52)	38 (76)	N/A
**Surgeon level of training, n (%)**		**N/A**	**N/A**	**.27**
	PGY1-3^b^	13 (26)	9 (18)	N/A
	PGY4-6^c^	30 (60)	8 (16)	N/A
	Fellow	3 (6)	8 (16)	N/A
	Attending	3 (6)	9 (18)	N/A
	Other	1 (2)	1 (2)	N/A
# Surgeons at site, mean (SD)		1.6 (0.7)	1.7 (0.6)	.87
# Surgeons in case, mean (SD)		2.7 (0.7)	2.9 (0.9)	.38
Length of incision in centimeters, mean (SD)		29.1 (1.7)	33.3 (2.2)	.13
Right-handed, n (%)		50 (100)	47 (94)	.08

^a^N/A: not applicable.

^b^PGY 1-3: postgraduate year 1-3.

^c^PGY4-6: postgraduate year 4-6.

While the distribution of case types differed significantly between control and intervention groups (Table 1, *P*=.02), breast reconstruction was the most frequent case type in both groups. All other surgeon and case characteristics, including surgeons’ level of training, number of surgeons per case, number of surgeons at each surgical site during a case, and mean length of incision did not significantly differ between control and intervention groups. Most surgeons in both groups were right-handed (15/15, or 100% in the control group vs 15/16, or 94% in the Operative Armour group).

### Control Versus Operative Armour Needle Use Outcomes

Across all 100 cases, 2234 needles were observed (1037 needles in control cases and 1197 needles in intervention cases). On average, users required 4.8 needles to acclimate to the use of Operative Armour (Table 2). The number of needles used per incision, stratified by case type, did not significantly differ between control and intervention groups.

**Table 2:** Needle use statistics for control and Operative Armour cohorts, stratified by case type.

Characteristic		Control (n=1037)	Operative Armour (n=1197)	*P* value
# Stitches for acclimation, mean (SD)		N/A^a^	4.8 (3.5)	N/A
**# Stitches used per incision, mean (SD)**		**N/A**	**N/A**	**N/A**
	Abdominal surgery	21.3 (14.4)	20.8 (7.1)	.93
	Breast reconstruction—breast site	20.1 (11.1)	24.4 (11.8)	.50
	Breast reconstruction—donor site	44.4 (17.2)	44.6 (19.9)	.34
	Mastopexy/breast revision	24.4 (11.8)	28.2 (9.1)	.54
**# Passes per stitch, mean (SD)**		**2.0 (0.3)**	**0 (0)**	**<.001**
	Abdominal surgery	2.0 (0)	0 (0)	<.001
	Breast reconstruction	2.0 (0.1)	0 (0)	<.001
	Mastopexy/breast revision	2.1 (0.6)	0 (0)	<.001
**# Needle adjustments per stitch, mean (SD)**		**0.2 (0.4)**	**0.1 (0.3)**	**.004**
	Abdominal surgery	0.1 (0.3)	0.1 (0.4)	.53
	Breast reconstruction	0.2 (0.4)	0.1 (0.3)	.03
	Mastopexy/breast revision	0.2 (0.4)	0.05 (0.2)	<.001
**# Needles waiting, mean (SD)**		**2.3 (1.0)**	**0.2 (0.5)**	**<.001**
	Abdominal surgery	1.9 (0.8)	0 (0)	<.001
	Breast reconstruction	2.7 (1.0)	0.2 (0.6)	<.001
	Mastopexy/breast revision	1.7 (0.8)	0.2 (0.4)	<.001
**Proportion of needles dropped, n/N (%)^b^**		**4/1037 (0.4)**	**2/1197 (0.1)**	**.32**
	Abdominal surgery	0/234 (0)	0/207 (0)	>.99
	Breast reconstruction	2/557 (10.4)	2/845 (0.2)	.34
	Mastopexy/breast revision	2/246 (8.1)	0/145 (0)	.28
**Proportion of needles temporarily misplaced, n/N (%)^b^**		**8/1037 (0.8)**	**4/1197 (0.3)**	**.11**
	Abdominal surgery	1/234 (0.4)	0/207 (0)	.36
	Breast reconstruction	4/557 (0.7)	2/845 (0.2)	.14
	Mastopexy/breast revision	3/246 (1.2)	2/145 (1.4)	.87
**Proportion of needles lost, n/N (%)^b^**		**0/1037 (0)**	**1/1197 (0.1)**	**.87**
	Abdominal surgery	0/234 (0)	0/207 (0)	>.99
	Breast reconstruction	0/557 (0)	0/845 (0)	>.99
	Mastopexy/breast revision	0/246 (0)	1/145 (0.7)	.11
**Proportion of needlesticks, n/N (%)^c^**		**1/50 (2)**	**0/50 (0)**	**.36**
	Abdominal surgery	1/11 (9)	0/8 (0)	.38
	Breast reconstruction	0/26 (0)	0/38 (0)	>.99
	Mastopexy/breast revision	0/13 (0)	0/4 (0)	>.99

^a^N/A: not applicable.

^b^Proportions reported out of total number of needles used.

^c^Proportion reported out of total number of cases.

Overall, Operative Armour led to significant decreases in the mean number of needle adjustments by hand per stitch (1 adjustment per every 5 stitches in control cases vs 1 adjustment per every 10 stitches in Operative Armour, or intervention, cases, *P*=.004). In fact, needle adjustments occurred half as frequently with use of the device in free flap breast reconstruction cases (1 in 5 stitches in control vs 1 in 10 stitches with intervention, *P*=.03) and one-fourth as frequently for mastopexy/breast revision cases (1 in 5 stitches in control cases vs 1 in 20 stitches in Operative Armour cases, *P*<.001). A multivariable linear regression established that intervention (Operative Armor) significantly decreased the number of needle adjustments by hand observed, after adjusting for surgeons’ level of training and for case type (*F*_4, 21.68_=3.72; 95% CI 1.82-5.98; *P*=.01).

### Control Versus Operative Armour Needle Safety Outcomes

Use of Operative Armour eliminated needle passing during skin closure (*P*<.001 compared with control cases). The mean number of loaded and unprotected waiting needles per case was also significantly decreased in the Operative Armour group (2.3 needles in control cases vs 0.2 in intervention cases, *P*<.001). However, there was no significant difference in overall proportion of needles dropped (41037, or 0.4% in control vs 2/1197, or 0.1% with Operative Armour; *P*=.32), temporarily misplaced (8/1037, or 0.8% in control vs 4/1197, or 0.3% with Operative Armour; *P*=.11), and permanently lost (0/1037, or 0% in control vs 1/1197, or 0.1% with Operative Armour; *P*=.87). Out of all 100 cases, one Operative Armour case required an x-ray to locate a lost needle (not found in the patient) and one NSI occurred in a control case; both findings did not represent a significant difference between groups (*P*=.36 for both).

### Post Hoc Power Analyses

Post hoc power analyses were conducted for all analyses. Based on the number of cases observed (50 control, 50 intervention) and the effect size with regard to needle use and safety observed between case and control study groups, we determined that our study had a power greater than 0.8 for all analyses conducted.

## Discussion

### Principal Findings

Observations of 50 control and 50 intervention surgeries using the Operative Armour device showed a significant decreased in the number of needle adjustments by hand with use of the device. Although no significant difference was seen in NSI incidence, the device eliminated needle passing during skin closure and decreased the number of loaded and unprotected waiting needles. No significant difference was observed in dropped, temporarily misplaced, or lost needles.

NSIs pose a significant occupational health risk over the course of a surgeon’s career. Given the potential for a needlestick to transmit an infectious blood borne disease such as HIV, in 2000, the Needlestick Safety and Prevention Act was signed into law, which required the US Occupational Safety and Health Administration to revise their Bloodborne Pathogens Standard to include additional requirements to prevent NSIs [9]. Under this revised standard, employers are required to identify and use engineered sharps injury prevention (ESIP) devices or devices that are engineered to have a higher level of safety [10-12]. As needlesticks are inversely related to experience and age, teaching hospitals have a particular responsibility to adopt devices and programs that may help prevent these occupational injuries [10,13].

Previously attempted tactics to decrease NSIs include double gloving [14] and creating a neutral zone on the surgical field in which sharps are placed, eliminating the direct hand-to-hand technique [15]. However, having contaminated needles on the field still elevates the risk of NSIs. Hitchhiker sharps, in which needles hitchhike with other instruments or materials (such as gauze), can still injure an unknowing party, even when left in a neutral zone [1]. Additionally, in cases in which there are multiple operative sites and numerous surgeons, it is challenging to identify an appropriate neutral zone location that is accessible for both scrub techs and surgeons. It is therefore unsurprising that although it is common practice to double glove, many institutions do not regularly employ any additional needlestick prevention protocols.

Safety-engineered devices are designed to improve safe handling of sharps by incorporating a built-in protection mechanism. Safety-engineered devices are used predominantly in nonsurgical settings and have been found to have mixed efficacy in reducing NSIs, with some studies finding that safety-engineered devices actually increase risk of these injuries [16,17]. In fact, a Cochrane review analyzing 24 studies investigating devices for preventing NSIs in nonsurgical settings reported uniformly low-quality evidence with inconsistent results [18]. ESIP devices are a class of safety-engineered devices that provide mechanical protection from sharp injuries. However, there are currently no studies to demonstrate the effectiveness of intraoperative ESIP devices in preventing needlestick or sharps injuries. Consequently, there is a need for more high-quality, controlled studies, especially in surgical settings.

To our knowledge, this study is the first to date to study and demonstrate clinical effectiveness of a wearable intraoperative ESIP device. First, although not significantly different, the only NSI that occurred during the conduction of this trial occurred during a control case. Second, as Operative Armour was not associated with any difference in dropped, temporarily misplaced, or lost needles, the device demonstrates noninferiority compared with current practices. Third, by minimizing sharps behaviors that drive NSIs such as the manipulation, handling, and passing of intraoperative sharps, Operative Armour demonstrates superiority over current practice in the potential to significantly decrease sharps injuries. Therefore, Operative Armour functions effectively as an intraoperative ESIP device.

In addition to efficacy, ease of device incorporation is also critical to assess for any new device. The attendings and residents involved in the study were initially given a training session outside of the operating room in which they were taught how to wear the device and secure needles within the holder. Each surgeon’s intraoperative acclimation period was also observed and averaged fewer than 5 needles, indicating surgeons were able to efficiently adjust to the device during the first case of its use. Interestingly, surgeons with more years of operative experience (eg, chief residents, fellows, and attendings) typically required a longer period of time to acclimate to the device when compared with younger surgeons (eg, junior residents). This could potentially represent the concept of conscious versus unconscious competence—as younger surgeons are still honing their basic technical suturing skills, they are likely more consciously competent during the conduction of these tasks. However, more experienced surgeons are unconsciously competent with skin closure, so introducing a new device may have required a temporary shift back to more mindful suturing practices.

In addition to the device’s impact on needlestick risk, it may also have the potential to improve operative efficiency by introducing parallel processing in which two or more separate processes are conducted simultaneously rather than in series [19-22]. When the surgery team is closing the wound at the end of a surgical case, the scrub tech and circulator nurse are usually kept occupied making sure the surgeons have the sutures and dressings needed to finish the case, including loading and unloading the needle driver each time a surgeon needs a new suture. Often, it is not until the surgery is finished that the scrub tech and circulator can do their final count and begin breaking down the room. However, if the scrub tech and circulator were free do those tasks while the surgeon is closing independently, those processes could therefore be conducted in parallel. Operative Armour may have the potential to facilitate this parallel processing by allowing the surgeon to manage their own sutures. When the surgeon is ready to close, the scrub tech can give the self-service device to the surgeon and then begin the final count as the surgeon has all the equipment needed to finish the case. Future studies could therefore look at the effect of Operative Armour on operative efficiency, ideally through the use of paired time measurements of same-surgeon same-facility closure times with and without the device, as well as overall turnover time. This analysis would in turn enable a more accurate cost/benefit ratio analysis of this device. Included procedures used an average of two Operative Armour barrier kits (US $130 each, including 1 barrier arm band and 6 needle holder pieces) for a total of US $260 per case. Future research may determine whether Operative Armour affects the financial considerations associated with operative efficiency, as well as costs associated with NSIs.

### Limitations

This study has a few limitations. First, due to the setting in an academic environment, the teams of circulator and scrub tech frequently varied. Although staff was typically familiar with plastic surgery cases, more familiarity with the device due to consistent staffing may have further affected needle handling and use of the device. As the trainees studied also moved on or off this service throughout the 4 months of observation, we were unable to do paired measurements of same-surgeon closure times with and without the device. However, as trainees in academic environments experience a higher risk for NSIs, this was the environment we were most interested in investigating. Last, although we were able to find statistically significant differences in behaviors that increase NSI risk, we were not powered to find a difference in NSIs due to the rarity of these occurrences.

This study is the first clinical review of an ESIP device to prevent NSIs in the operating room. By addressing needle passing and handling—key contributors to intraoperative NSIs—Operative Armour has the potential to decrease the risk of NSIs for both surgeons and perioperative staff. These findings are especially important for academic teaching hospitals to consider as younger practitioners are at the highest risk for NSIs. Further research may be done to identify the impact of this device on operative efficiency as well.

### Conclusions

Operative Armour effectively functions as an ESIP device by decreasing intraoperative needle passing and handling. Although sample size prohibits demonstrating a decrease in NSIs during observed cases, by decreasing behaviors that drive NSI risk, we anticipate an associated decrease in NSIs with use of the device. By decreasing these injuries, we can safeguard the health of health care workers at all levels, from attending to nurse to medical student.

## Abbreviations

ESIP: engineered sharps injury prevention
NSI: needlestick injury
